# Supplementary material for: Ecotype Division and Chemical Diversity of Cynomorium songaricum from Different Geographical Regions
Source: Molecules. 2022 Jun 21;27(13):3967. doi: 10.3390/molecules27133967 (PMC9268089; doi:10.3390/molecules27133967)
Supplement: Supplementary file 1 [file molecules-27-03967-s001.zip › Supplementary_materials.pdf]

V
B
B
B
B
B
B
B
B
B

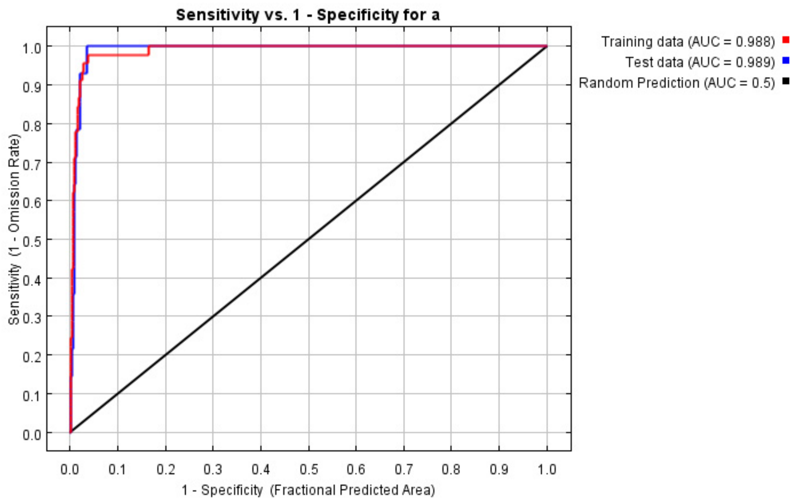

Figure S1 The AUC using ROC methods for testing the result of Regularized training gain.

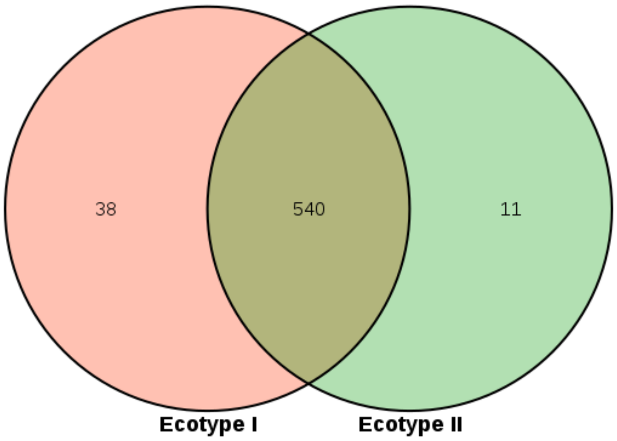

Figure S2 Venn diagrams of total metabolites among two ecotypes.
